# New Appliances

**Published:** 1911-10-07

**Authors:** 


### NEW APPLIANCES.

### RUBBER APPLIANCES.

The catalogue of Messrs. Anderson, Anderson and Anderson, 37 Queen Victoria Street, contains a varied list of those articles now made in rubber for institution purposes which were not thought of as possible some years ago. The problem of rubber sheeting that shall resist corrosive substances is still with us, but among this firm's goods can be found a number designed for such hospital purposes. Hot-water bottles, bedpans, sheeting, and mackintosh are included to meet different needs, and now the varied uses of rubber are known it is not for novelties so much as for reliability of quality that hospital works now look, especially in view of rubber's tendency to advance in price.

### A NON-CRACKING WALL-PAINT.

The Japan paint known as "Velure," and manufactured by C. Chancellor and Co., 13 Clerkenwell Road, London, claims to be non-cracking, and if any opinion can be based on a coated sample of metal which can be bent in all directions while remaining intact, this claim is certainly made good. Many hospitals have tried it, both for inside and outside painting, and the result of inquiries shows that the extra cost of material and labour involved by a varnish paint, which is stiffer to wash than other paints, is paid for by durability. Hospital committees therefore find it exceedingly easy to obtain the experience of their colleagues, the number of which shows it to have a deserved reputation.

### CAMWAL TABLE-WATERS.

The samples of these table-waters we have received from Camwal, Ltd., 112 Pembroke Street, London, N., included bottles of seltzer water, stone-ginger beer, lemonade, and syphons of lithia, soda, and potash waters. Analysis proves these waters to be what they are represented—pure table-waters containing appreciable amounts of salts. The stone-ginger beer is especially good, possessing distinct body and flavour. Similarly the lemonade has a crisp fruity flavour which makes it an agreeable and stimulating drink. The lithia, soda, and potash waters are slightly alkaline, and may claim to be definite saline waters of a very distinct sparkling nature, the aeration being exceptionally good, so that the waters retain their sparkle for a comparatively long time. The Camwal brand is a sure sign of excellence, and we can safely recommend these waters to medical men and institutional workers who desire to have a first-class artificial table-water at a low price.

